# Association between novel adiposity parameters and hyperuricemia: a cross-sectional study

**DOI:** 10.3389/fnut.2025.1536893

**Published:** 2025-03-26

**Authors:** Baoan Wang, Chuncheng Ma, Jinhua Wu, Ze Huang

**Affiliations:** ^1^Affiliated Jiangmen TCM Hospital of Ji’nan University, Jiangmen, Guangdong, China; ^2^Jiangmen Wuyi Hospital of Traditional Chinese Medicine, Jiangmen, Guangdong, China

**Keywords:** cross-sectional study, adiposity parameters, hyperuricemia, National Health and Nutrition Examination Survey, body roundness index

## Abstract

**Objectives:**

Body mass index (BMI) is a commonly used parameters to measure obesity, but it cannot well reflect the distribution of body fat, which has limitations in clinical practice. Novel adiposity parameters have emerged as substitution to BMI to assess obesity. This study aimed to comprehensively investigate the association between hyperuricemia and novel adiposity parameters.

**Methods:**

We included data from the National Health and Nutrition Examination Survey from 1999–2006. Weighted logistic regression was employed to evaluate the relations between hyperuricemia and novel adiposity parameters, including body roundness index (BRI), weight-adjusted waist index (WWI), a body shape index (ABSI), and conicity index (CoI). To assess the most diagnostic factor for hyperuricemia, the receiver operating characteristic (ROC) curve analysis was employed. The area under the curve (AUC) was used to assess the diagnostic power of each parameter.

**Results:**

The study included 24,763 participants, 3,528 of whom were diagnosed with hyperuricemia. Compared with the first quartile (Q1), the fourth quartile’s (Q4) BRI, WWI, ABSI and CoI were linked to an increased risk of hyperuricemia (OR: 9.34, 95% CI: 7.73–11.28; OR: 4.67, 95% CI: 3.97–5.49; OR: 2.61, 95% CI: 2.26–3.02; OR: 7.34, 95% CI: 6.12–8.81, respectively). This relationship persisted after adjusting for confounding factors. Among the four novel obesity parameters, BRI had the largest AUC and was a good diagnostic index of hyperuricemia (AUC = 0.697 for male and AUC = 0.751 for female).

**Conclusion:**

In the general population, larger obesity parameters are linked to a higher risk of hyperuricemia. BRI has high diagnostic value and can be used as a new index for the evaluation of hyperuricemia. This study provides a new basis for the prevention and monitoring of hyperuricemia.

## 1 Introduction

Hyperuricemia refers to a state where blood uric acid (UA) concentrations are abnormally high, typically exceeding 420 μmol/L in males and 360 μmol/L in females (1). With changes in lifestyle and diet, the prevalence of hyperuricemia has been escalating annually, posing a significant global public health challenge (2). Recent epidemiological investigations have revealed that the prevalence of hyperuricemia in Western countries, such as the United States, reaches as high as 20% (3), while in China, it varies between 8.4 and 25% among adults (4). Purines in food are broken down into hypoxanthine and xanthine during digestion, ultimately converting to UA (5). The reactive oxygen species generated alongside UA can promote metabolic disorders, suggesting that hyperuricemia may influence the development of systemic diseases (5). Serum uric acid levels have been associated with heart disease, hypertension, arteriosclerosis, and osteoarthritis (6–9). Moreover, hyperuricemia often coexists with other metabolic syndrome features, like obesity and insulin resistance, which further heightens health risks (10, 11). Therefore, identifying the underlying factors associated with hyperuricemia is essential for alleviating pressure on the healthcare system, enhancing patient care quality, and developing new treatment strategies.

Obesity leads to a series of changes, such as excess fat accumulation, insulin resistance, and reduced excretion of UA by the kidneys, which creates an environment where too much UA is produced and not enough is excreted, ultimately increasing the risk of hyperuricemia (12, 13). Traditional parameters of adiposity, such as body mass index (BMI), waist circumference (WC) and waist-to-height ratio (WHtR), are commonly employed in clinical settings and public health initiatives (14). BMI is a measure of overall obesity, failing to discern the specific distribution of body fat or differentiate between subcutaneous and visceral fat deposits (15, 16). The use of BMI has been somewhat limited by the existence of an “obesity paradox,” in which a higher BMI is associated with reduced mortality (17). WC and WHtR are sensitive measures of abdominal fat accumulation and are often used to assess central obesity, but their main shortcoming is that they ignore the height and weight of the subject (18, 19). Additionally, due to the limitations of traditional obesity metrics in assessing body shape and fat distribution, researchers have proposed new obesity indicators to differentiate between obesity patterns, such as body roundness index (BRI) (20), weight-adjusted waist index (WWI) (21), a body shape index (ABSI) (22), and conicity index (CoI) (23). A cohort study conducted in a rural region of China revealed a significant correlation between BRI and ABSI with hyperuricemia, with BRI emerging as a more diagnostic parameter of hyperuricemia compared to ABSI (24). However, fewer studies have comprehensively assessed the association between different novel adiposity parameters and hyperuricemia.

In this large population-based cross-sectional investigation, we aimed to comprehensively explore the relationship between four novel adiposity parameters (BRI, WWI, ABSI, and CoI) and hyperuricemia.

## 2 Methods

### 2.1 Data source

The National Health and Nutrition Examination Survey (NHANES) is a comprehensive initiative designed to assess the health and nutritional profiles of the U.S. population. Utilizing a multifaceted methodology that encompasses interviews, physical examinations, and laboratory analyses, NHANES collects data on a wide array of health conditions, lifestyle factors, and dietary practices. Its cross-sectional design, paired with representative sampling techniques, enables thorough exploration of public health trends and risk factors among various demographic groups.

### 2.2 Population selection

**Figure 1:**
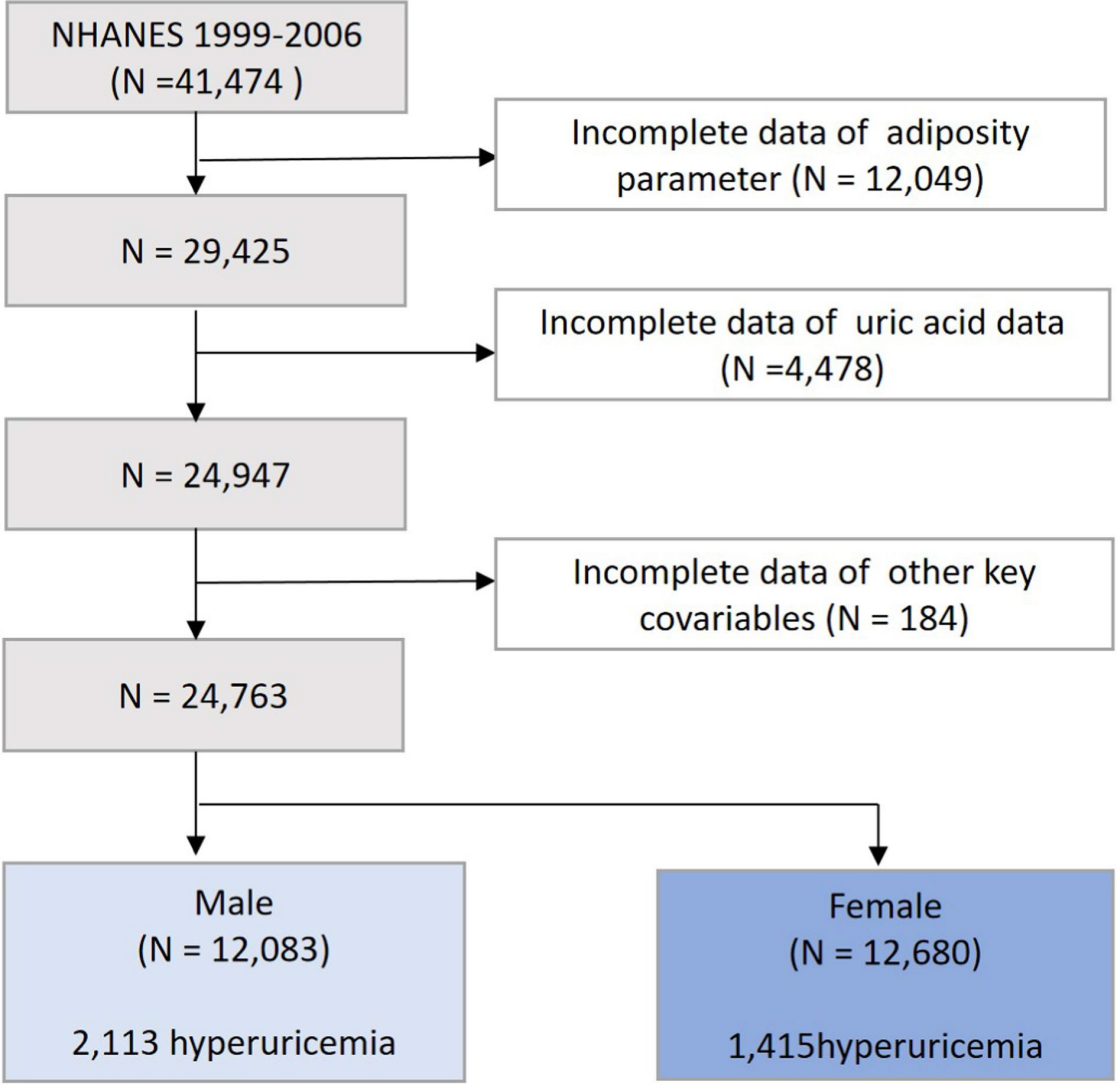
Flowchart of participants.

We selected data from 4 interview cycles of NHANES from 1999 to 2006, with a total of 41,474 participants surveyed. Populations lacking the data on the adiposity parameters (*n* = 12,049), uric acid (*n* = 4,478), and key covariates (*n* = 184) were further excluded. Ultimately, our study included 24,763 participants, comprising 12,083 men and 12,680 women (Figure 1).

### 2.3 Measurement of adiposity parameters

The participant stood barefoot, aligned with a vertical stadiometer plate for height measurement. Body weight was assessed using a calibrated digital scale, with participants wearing minimal clothing. WC was determined with participants standing in a relaxed posture, measuring the distance between the iliac crest and the lower rib margin. All measurements were conducted according to standardized procedures to ensure accuracy and reliability. The adiposity parameter was calculated using the following formula:


$$BRI=364.2–365.5\times \sqrt{1-\left(\frac{{\left(WC\left(m\right)/2\pi \right)}^{2}}{{\left(0.5\times \text{Height}\left(m\right)\right)}^{2.}}\right)}$$

$$WWI=\;\frac{WC\;\left(\text{cm}\right)}{\sqrt{\;\text{Weight}\;\left(\text{kg}\right)}}$$

$$\text{ABSI}=\frac{WC\left(m\right)}{{BMI}^{2/3}\text{Height}{\left(m\right)}^{1/2}}\times 1000$$

$$CoI=\frac{{WC}_{\left(m\right)}}{0.109\times \sqrt{\frac{{\text{Weight}}_{\left(\text{kg}\right)}}{{\text{Height}}_{\left(m\right)}}}}$$

### 2.4 Definition of hyperuricemia

Serum UA concentrations were measured utilizing the Roche Cobas 6,000 chemistry analyzer (Roche Diagnostics, Indianapolis, IN 46,250). During the assay, UA is catalyzed by uricase, reacting with allantoin and hydrogen peroxide (H₂O₂) to generate a measurable signal. Hyperuricemia is characterized by a serum UA level of ≥420 μmol/L in males and ≥ 360 μmol/L in females.

### 2.5 Assessment of covariates

Demographic characteristics were collected through standardized questionnaires, including variables such as age, degree of education, gender, race/ethnicity, smoking status, alcohol intake, and comorbid health conditions. Educational levels were stratified into three categories: higher education (college or above), some college or equivalent, and high school or below. Alcohol consumption was categorized into three distinct groups: non-drinker, moderate-to-heavy drinker, and light drinker. Smoking is classified into three conditions: those who have never smoked, former smokers, and current smokers. The definition of hypertension adheres to the specified criteria: a systolic blood pressure of 140 mmHg or higher, a diastolic blood pressure of 90 mmHg or higher (25); ongoing taking antihypertensive medications; or a self-reported diagnosis of hypertension. The diagnosis of diabetes mellitus was established based on the specified criteria: (1) HbA1c ≥ 6.5%, fasting plasma glucose ≥7.0 mmol/L, or plasma glucose ≥11.1 mmol/L following a 2-h oral glucose tolerance test (26); (2) self-reported history of diabetes; or (3) currently taking antidiabetic drugs.

### 2.6 Statistical analysis

Categorical variables are expressed by quantity and percentage (%), and continuous variables are expressed by median (25-75th percentile). The Chi-square test and Wilcoxon rank test were used to compare the differences between different groups. Four parameters were divided into quartiles (Q1–Q4), with Q1 as the benchmark group for comparison. Weighted logistic regression model was used to evaluate the relationship between obesity parameters and hyperuricemia, and the odds ratio (OR) and 95% confidence interval (CI) were calculated. Single-factor and multiple-factor regression models were established. Model 1 is a crude model without adjusting for confounding factors. Model 2 adjusted for race, gender, education level and age; Model 3 adjusted for alcohol consumption, smoking status, hypertension, and diabetes on the basis of Model 2. The receiver operating characteristic (ROC) curve was used to evaluate the effectiveness of different parameters in patients with hyperuricemia. All data were analyzed using Stata software (version 18.1, Stata Corp. LP, College Station, Texas, United States), and *p* < 0.05 was considered statistically significant.

## 3 Results

### 3.1 Baseline characteristics

Of the 24,763 participants, the median age was 33 years (interquartile range: 18–55 years) and the number of people with hyperuricemia was 3,528 (14.25%). The prevalence of hyperuricemia was higher in men than in women (59.9% vs. 40.1%). Compared to those with normal UA levels, those with hyperuricemia were older, less educated, and had a higher rate of comorbid hypertension. In addition, those with hyperuricemia had higher BMI and WC levels (Table 1).

**Table 1:** Characteristics of study participants included in the analyses of the 1999–2006 datasets.

Characteristic	Total (*N* = 24,763)	Hyperuricemia (−) (*N* = 21,235)	Hyperuricemia (+) (*N* = 3,528)	*P*-value
Age (years)	33 (18, 55)	31 (17, 52)	49 (25, 68)	<0.001
Gender				<0.001
Male	12,083 (48.8%)	9,970 (47.0%)	2,113 (59.9%)	
Female	12,680 (51.2%)	11,265 (53.0%)	1,415 (40.1%)	
Ethnicity, n (%)				<0.001
Mexican American	6,546 (26.4%)	5,864 (27.6%)	682 (19.3%)	
Other Hispanic	1,012 (4.1%)	895 (4.2%)	117 (3.3%)	
Non-Hispanic White	10,513 (42.5%)	8,787 (41.4%)	1,726 (48.9%)	
Non-Hispanic Black	5,756 (23.2%)	4,904 (23.1%)	852 (24.1%)	
Other race	936 (3.8%)	785 (3.7%)	151 (4.3%)	
Education level, n (%)				<0.001
≤ High school	8,997 (54.1%)	7,370 (53.5%)	1,627 (57.0%)	
College	4,431 (26.6%)	3,681 (26.7%)	750 (26.3%)	
> College	3,202 (19.3%)	2,726 (19.8%)	476 (16.7%)	
Smoking, n (%)				<0.001
Current	3,025 (18.9%)	2,585 (19.5%)	440 (16.0%)	
Former	4,377 (27.4%)	3,427 (25.9%)	950 (34.6%)	
Never	8,589 (53.7%)	7,232 (54.6%)	1,357 (49.4%)	
Drinking, n (%)				<0.001
Never	4,260 (41.4%)	3,440 (40.1%)	820 (48.2%)	
Mild	2,545 (24.7%)	2,193 (25.6%)	352 (20.7%)	
Moderate or Heavy	3,480 (33.8%)	2,950 (34.4%)	530 (31.1%)	
Diabetes mellitus, n (%)				<0.001
Yes	22,903 (92.5%)	19,834 (93.4%)	3,069 (87.0%)	
No	1,860 (7.5%)	1,401 (6.6%)	459 (13.0%)	
Hypertension, n (%)				<0.001
Yes	6,704 (27.1%)	4,947 (23.3%)	1,757 (49.8%)	
No	18,059 (72.9%)	16,288 (76.7%)	1,771 (50.2%)	
Adiposity parameters				
BRI	4.281 (2.914, 5.819)	4.045 (2.766, 5.551)	5.637 (4.409, 7.131)	<0.001
WWI	10.688 (10.025, 11.33)	10.609 (9.961, 11.254)	11.102 (10.541, 11.654)	<0.001
ABSI	0.079 (0.076, 0.083)	0.079 (0.075, 0.083)	0.081 (0.078, 0.085)	<0.001
CI	1.264 (1.185, 1.341)	1.253 (1.176, 1.330)	1.327 (1.262, 1.384)	<0.001

Continuous and categorical variables are expressed in medians (25th–75th percentile) and numbers (percentages). BRI, body roundness index; WWI, weight-adjusted waist index; ABSI, body adiposity index; CI, conicity index.

### 3.2 Relationship between novel adiposity parameters and hyperuricemia

**Figure 2:**
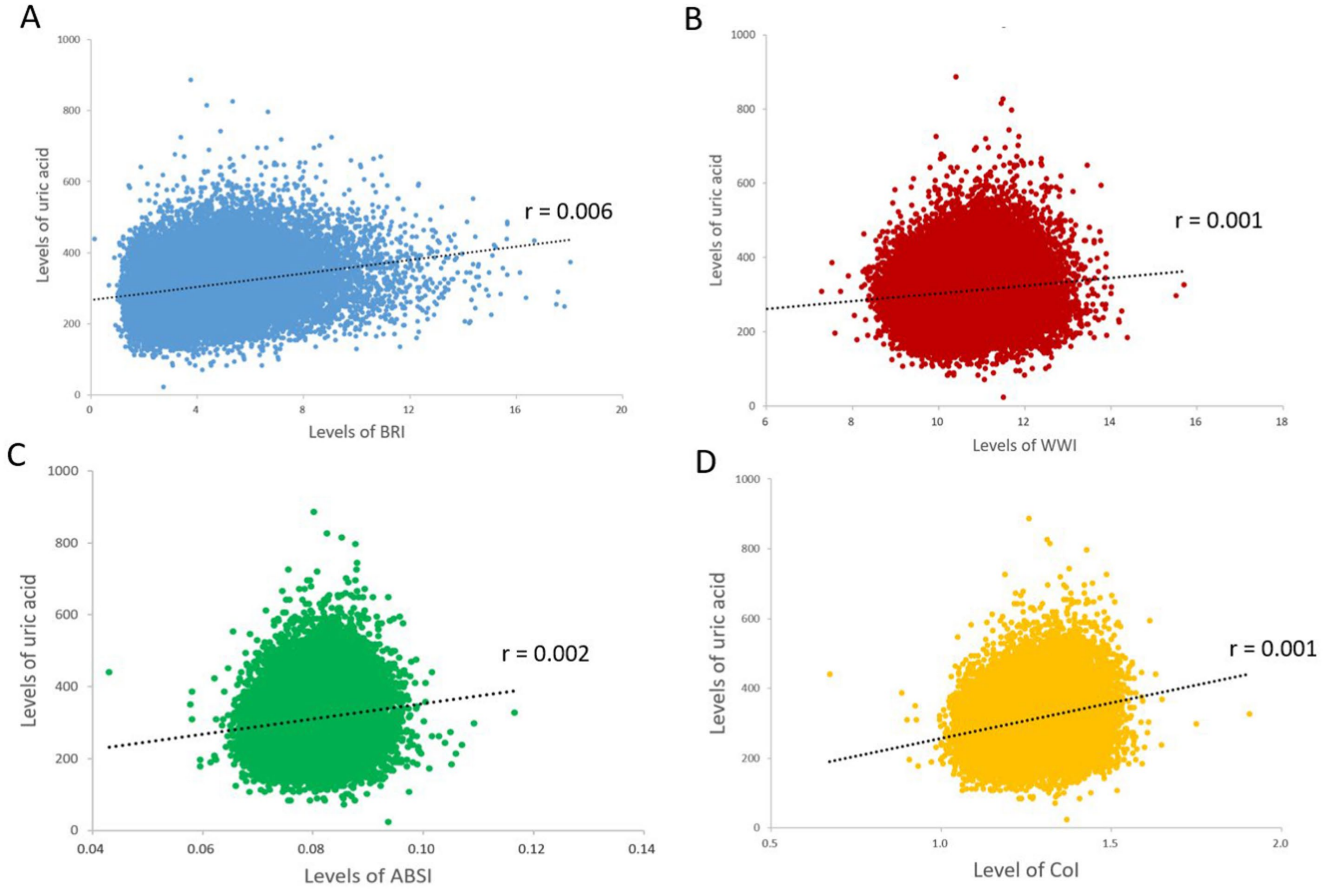
Heat maps to evaluate the association between novel obesity parameters and uric acid. Correlation coefficients (r) were used to represent the association between novel metabolic parameters and uric acid levels. **(A)** BRI, uric acid; **(B)** WWI, uric acid; **(C)** ABSI, uric acid; **(D)** CoI, uric acid. BRI, body roundness index; WWI, weight-adjusted waist index; ABSI, body adiposity index; CoI, conicity index.

Figure 2 presents a heat map between novel obesity parameters and hyperuricemia. We observed that four novel obesity parameters were positively associated with the risk of hyperuricemia (all *p* values were < 0.05). Compared to Q1, BRI (OR: 9.34, 95% CI: 7.73–11.28), WWI (OR: 4.67, 95% CI: 3.97–5.49), ABSI (OR: 2.61, 95% CI: 2.26–3.02), and CoI (OR: 7.34, 95% CI: 6.12–8.81) in the Q4 group developed a significantly increased risk of hyperuricemia. This association remained valid after adjusting for confounders (Table 2).

**Table 2:** Correlation between adiposity parameters and hyperuricemia.

	Model 1	Model 2	Model 3
BRI			
Q2 vs. Q1	2.70 (2.20, 3.31)	2.67 (2.01, 3.54)	3.04 (2.15, 4.30)
Q3 vs. Q1	5.13 (4.23, 6.23)	4.62 (3.51, 6.08)	4.93 (3.51, 6.93)
Q4 vs. Q1	9.34 (7.73, 11.28)	8.92 (6.8, 11.70)	9.26 (6.59, 13.02)
P for trend	<0.001	<0.001	<0.001
WWI			
Q2 vs. Q1	1.96 (1.64, 2.33)	1.99 (1.60, 2.48)	2.01 (1.54, 2.62)
Q3 vs. Q1	3.15 (2.67, 3.72)	2.97 (2.39, 3.68)	3.02 (2.32, 3.94)
Q4 vs. Q1	4.67 (3.97, 5.49)	4.46 (3.58, 5.56)	4.33 (3.27, 5.73)
P for trend	<0.001	<0.001	<0.001
ABSI			
Q2 vs. Q1	1.37 (1.17, 1.60)	1.22 (1.02, 1.47)	1.21 (0.96, 1.54)
Q3 vs. Q1	2.02 (1.74, 2.34)	1.51 (1.26, 1.81)	1.42 (1.13, 1.79)
Q4 vs. Q1	2.61 (2.26, 3.02)	1.56 (1.29, 1.89)	1.56 (1.22, 1.99)
P for trend	<0.001	<0.001	<0.001
CI			
Q2 vs. Q1	2.42 (1.98, 2.95)	2.46 (1.88, 3.23)	2.74 (1.96, 3.84)
Q3 vs. Q1	4.60 (3.81, 5.54)	4.16 (3.20, 5.39)	4.25 (3.05, 5.91)
Q4 vs. Q1	7.34 (6.12, 8.81)	5.98 (4.59, 7.80)	6.20 (4.42, 8.69)
P for trend	<0.001	<0.001	<0.001

The data is represented by OR and 95% CI. Model 1: no covariates were adjusted. Model 2: age, sex, race/ethnicity, and level of education were adjusted. Model 3: age, sex, race/ethnicity, level of education, smoking status, alcohol Consumption status, hypertension, and diabetes were adjusted. BRI, body roundness index; WWI, weight-adjusted waist index; ABSI, body adiposity index; CI, conicity index.

### 3.3 ROC curve analysis

**Figure 3:**
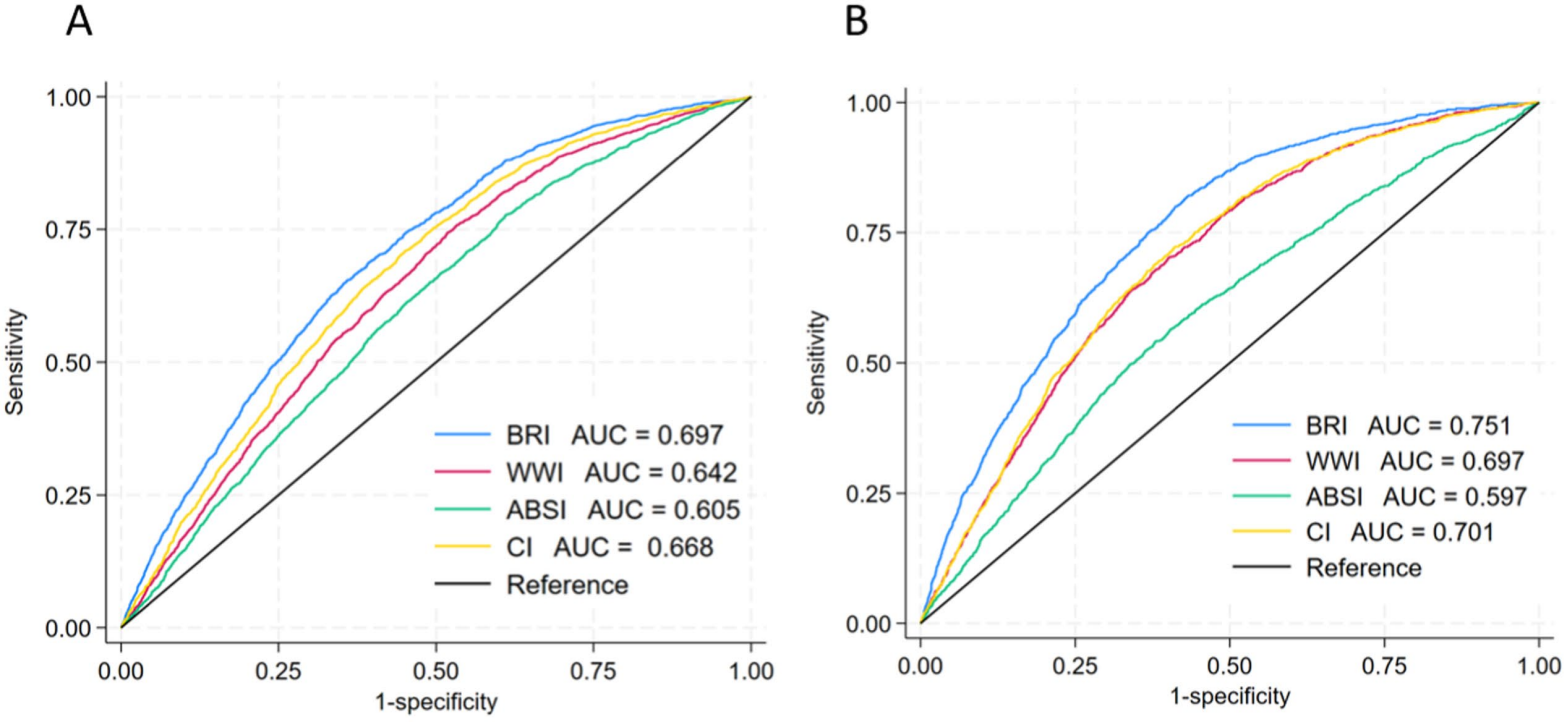
ROC curves were used to assess the association between obesity parameters and hyperuricemia. **(A)** In the male population. **(B)** In the female population. BRI, body roundness index; WWI, weight-adjusted waist index; ABSI, body adiposity index; CI, conicity index; AUC, area under curve.

We performed ROC curve analysis, with the area under the curve (AUC) representing diagnostic power. Among the four novel obesity parameters, BRI had the largest AUC and was a good diagnostic index of hyperuricemia (AUC = 0.697 for male and AUC = 0.751 for female) (Figure 3). This suggests that BRI is the best parameter for the diagnosis of hyperuricemia.

### 3.4 Subgroup analysis

We performed subgroup analyses of different genders (males and females), age groups (> 60 and ≤ 60 years), blood pressure status (with and without hypertension) and glycaemic status (with and without diabetes). The results showed that most of the obesity parameters were associated with an increased risk of hyperuricemia in the different subgroups. Of note, we did not observe a significant correlation between ABSI and the risk of hyperuricemia in women, age > 60 years, or in the hypertensive population (Supplementary Tables 1–4).

## 4 Discussion

In this population-based cross-sectional research, we found a correlation between an increase in four novel obesity parameters and an elevated risk of hyperuricemia, independent of demographic characteristics, lifestyle habits, hypertension and diabetes. ROC curve showed that BRI was the best parameter for the diagnosis of hyperuricemia in the US population.

Although BMI is indeed a widely employed and straightforward tool for assessing obesity in clinical practice and has been demonstrated to link to an elevated likelihood of developing hyperuricemia (27), its limitations are also of increasing concern. Obesity presents with a multifaceted phenotype, and while BMI is an indicator of overall obesity, it fails to accurately quantify the fat content of a particular area of the body. Evidence from Japan suggests that normal BMI with abdominal obesity increases the risk of hyperuricemia by approximately 40% compared to normal BMI with normal WC (28). Another study has also shown that waist circumference is a more reliable and unique indicator than BMI in the diagnosis of hyperuricemia (29).

The BRI, proposed by Thomas and colleagues, was initially utilized to assess the proportion of visceral fat and body fat (20). BRI combines the advantages of height eccentricity and WC to assess the health status of a population (30). A study of 124 people with prediabetes or non-medicated diabetes demonstrated a notable positive association between UA and BRI (31). Similar findings were reported in another study, which identified a dose–response relation between the BRI and hyperuricemia, indicating a 26% rise in the risk of hyperuricemia for each unit increase in BRI (32). Our results show that among the four novel adiposity parameters (BRI, WWI, ABSI, and CoI), BRI has the largest AUC and is the best diagnostic parameter of hyperuricemia. The ABSI calculation combined BMI and several BRI variables, which, like BRI, are parameters that reflect abdominal obesity (33). A cross-sectional study conducted by Anto et al. demonstrated that the BRI was significantly linked to metabolic syndrome after fully controlling for confounding variables, while ABSI was not (34). Another study has also shown that BRI has greater diagnostic power than ABSI when assessing metabolic disorders in children and adults (35, 36). The results of our subgroup analysis revealed no significant relations between ABSI and the risk of hyperuricemia in women, those aged >60 years, or those with hypertension. In addition, the value of the AUC is low (AUC = 0.605 for male and AUC = 0.597 for female), so ABSI may not be a good diagnostic parameter of hyperuricemia.

WWI is calculated based on standardized WC and body weight and is used to assess morbidity and mortality from obesity-related diseases. The WWI assesses body fat and muscle mass to provide a comprehensive view of body composition. An observational study including 602 older adults subjects revealed a positive association between WWI and visceral fat area (correlation coefficient: 0.262), as well as a negative association between WWI and muscle tissue (correlation coefficient: −0.511) (37). Another investigation has also demonstrated a stable positive relation between WWI and serum UA in adults over 20 years of age (38). CoI has been established as a diagnostic tool for patients with hypertension or diabetes (39). According to Nkwana and colleagues, CoI is not only significantly correlated with the risk of hypertension but also serves as a diagnostic indicator for fasting insulin levels and lipid profiles (40). Nevertheless, the relationship between CoI and hyperuricemia is not well-defined. Our findings suggest that an elevated CoI is significantly linked to an increased likelihood of hyperuricemia.

**Figure 4:**
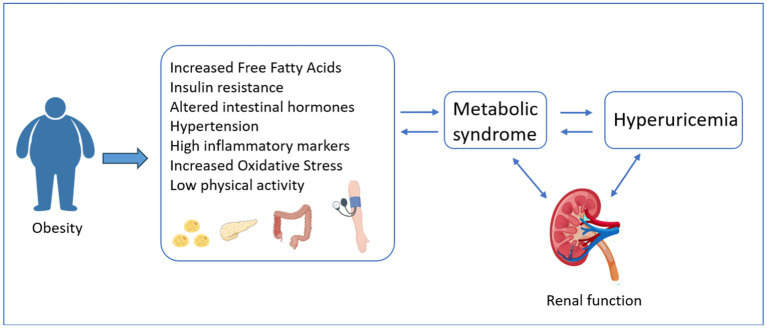
Mechanisms of obesity-induced hyperuricemia.

The pathological mechanism underlying the relationship between obesity and hyperuricemia remains incompletely understood, but it may be intricately linked to various factors such as: Firstly, in the context of obesity, the excessive accumulation of adipose tissue, particularly visceral fat, gives rise to insulin resistance (IR). IR, in turn, augments the activity of xanthine oxidoreductase, thereby stimulating the production and secretion of UA. Consequently, this cascade of events culminates in the manifestation of hyperuricemia (41). In addition, persistently high UA levels can induce and worsen IR by reducing glucose uptake and inhibiting nitric oxide production (42, 43). IR increases the level of insulin in the body, which in turn promotes peripheral and hepatic fat synthesis and inhibits lipolysis, thereby aggravating obesity (44). Changes in gut hormones responsible for appetite and glucose metabolism, such as glucagon-like peptide-1 (GLP-1), also affect insulin sensitivity (45). Secondly, obesity is known to activate the renin-angiotensin-aldosterone system, a cascade that regulates blood pressure and fluid balance. This activation can lead to vasoconstriction, which narrows blood vessels and increases blood pressure. Additionally, it promotes water and sodium retention, resulting in increased fluid volume in the body (46). These effects can have significant implications for renal excretion of UA. As the kidneys struggle to maintain homeostasis in the face of these changes, UA excretion may be impaired, leading to higher serum UA levels and further contributing to the development and progression of hyperuricemia. Thirdly, obesity increases the content of cytokines and adipokines, such as tumor necrosis factor-*α* (TN-α), interleukin-6 (IL-6), and monocyte chemoattractant protein-1 (MCP-1), which lead to widespread inflammation and are associated with oxidative stress (47, 48). The detailed mechanism can be found in Figure 4.

## 5 Strengths and limitations

This research is the first to comprehensively evaluate the relation between various adiposity parameters and the risk of hyperuricemia in the general population. It offers new insights into the specific mechanisms linking obesity and hyperuricemia, providing a foundation for future research. Additionally, by utilizing the nationally representative NHANES database, we minimized potential biases and enhanced the reliability of the findings.

This study presents several limitations. First, due to its cross-sectional design, it does not allow for the establishment of a causal relationship between the four adiposity parameters and hyperuricemia. Second, despite rigorous adjustment for known confounding variables inclusive of sex, education, and smoking habits, the possibility of residual confounding persists, which could potentially undermine the robustness of our findings. Third, our study sample was limited to older adults from the United States, necessitating a cautious approach when extrapolating the generalizability of our findings to disparate populations across different countries or age demographics.

## 6 Clinical implication

Persistent hyperuricemia can harm joint and kidney health and increase the likelihood of developing metabolic disorders, dementia, cardiovascular disease, and mortality (6, 49). Therefore, it is clinically important to establish early indicators of hyperuricemia and develop appropriate preventive strategies. In this population-based observational study, we observed that larger obesity parameters were associated with an increased risk of hyperuricemia, suggesting that they may serve as diagnostic indicators of hyperuricemia. These findings provide new insights into clinical practice and public health. Future well-structured cohort research is essential to clarify the mechanisms underlying the association between various obesity parameters and hyperuricemia.

## 7 Conclusion

In conclusion, in the general population, greater obesity parameters are associated with an increased risk of hyperuricemia. BRI has the largest AUC and can be used as a novel parameter for the diagnosis of hyperuricemia. This study offers new evidence for the prevention and monitoring of hyperuricemia.

## Data Availability

The original contributions presented in the study are included in the article/Supplementary material, further inquiries can be directed to the corresponding author.
