# Supplementary material for: Association between novel adiposity parameters and hyperuricemia: a cross-sectional study
Source: Front Nutr. 2025 Mar 26;12:1536893. doi: 10.3389/fnut.2025.1536893 (PMC11978630; doi:10.3389/fnut.2025.1536893)
Supplement: Supplementary file 1 [file Table_1.docx]

Supplementary Table 1: The association between adiposity parameters and hyperuricemia stratifed by gender (male and female).

|  | Male | Female |
| --- | --- | --- |
| **BRI** |  |  |
| Q2 vs Q1 | 3.05 (2.03, 4.57) | 3.33 (1.65, 6.69) |
| Q3 vs Q1 | 4.93 (3.31, 7.36) | 6.19 (3.13, 12.23) |
| Q4 vs Q1 | 8.15 (5.40, 12.31) | 14.88 (7.62, 29.06) |
| **WWI** |  |  |
| Q2 vs Q1 | 2.21 (1.63, 3.00) | 2.02 (1.14, 3.56) |
| Q3 vs Q1 | 3.44 (2.52, 4.70) | 3.50 (2.02, 6.07) |
| Q4 vs Q1 | 4.63 (3.24, 6.61) | 5.33 (3.09, 9.20) |
| **ABSI** |  |  |
| Q2 vs Q1 | 1.75 (1.24, 2.45) | 0.77 (0.54, 1.10) |
| Q3 vs Q1 | 1.93 (1.39, 2.68) | 1.14 (0.82, 1.60) |
| Q4 vs Q1 | 2.56 (1.79, 3.65) | 1.08 (0.77, 1.52) |
| **CI** |  |  |
| Q2 vs Q1 | 2.57 (1.65, 4.01) | 2.91 (1.72, 4.91) |
| Q3 vs Q1 | 4.40 (2.86, 6.77) | 4.06 (2.40, 6.87) |
| Q4 vs Q1 | 7.04 (4.51, 10.98) | 6.00 (3.55, 10.14) |

The data is represented by OR and 95% CI.

The model adjusted for age, race/ethnicity, level of education, smoking status, alcohol Consumption status, hypertension, and diabetes.

BRI, body roundness index; WWI, weight-adjusted waist index; ABSI, body adiposity index; CI, conicity index.

Supplementary Table 2: The association between adiposity parameters and hyperuricemia stratifed by age (age＞60 years and age ≤ 60 years ).

|  | Age＞60 years | Age ≤ 60 years |
| --- | --- | --- |
| **BRI** |  |  |
| Q2 vs Q1 | 3.37 (1.33, 8.49) | 2.95 (2.04, 4.27) |
| Q3 vs Q1 | 4.89 (1.98, 12.06) | 4.96 (3.45, 7.13) |
| Q4 vs Q1 | 9.26 (3.77, 22.77) | 9.63 (6.68, 13.89) |
| **WWI** |  |  |
| Q2 vs Q1 | 2.55 (1.03, 6.33) | 1.95 (1.49, 2.57) |
| Q3 vs Q1 | 4.14 (1.75, 9.76) | 2.93 (2.22, 3.86) |
| Q4 vs Q1 | 5.10 (2.19, 11.90) | 4.46 (3.28, 6.06) |
| **ABSI** |  |  |
| Q2 vs Q1 | 1.00 (0.51, 1.95) | 1.19 (0.93, 1.53) |
| Q3 vs Q1 | 1.47 (0.80, 2.68) | 1.35 (1.06, 1.74) |
| Q4 vs Q1 | 1.58 (0.88, 2.85) | 1.58 (1.21, 2.07) |
| **CI** |  |  |
| Q2 vs Q1 | 2.79 (1.23, 6.33) | 2.64 (1.83, 3.80) |
| Q3 vs Q1 | 2.77 (1.27, 6.04) | 4.23 (2.96, 6.04) |
| Q4 vs Q1 | 4.91 (2.28, 10.58) | 6.07 (4.20, 8.75) |

The data is represented by OR and 95% CI.

The model adjusted for sex, race/ethnicity, level of education, smoking status, alcohol Consumption status, hypertension, and diabetes.

BRI, body roundness index; WWI, weight-adjusted waist index; ABSI, body adiposity index; CI, conicity index.

Supplementary Table 3: The association between adiposity parameters and hyperuricemia stratifed by blood pressure status (with and without hypertension).

|  | Hypertension (+) | Hypertension (-) |
| --- | --- | --- |
| **BRI** |  |  |
| Q2 vs Q1 | 4.18 (2.18, 8.04) | 2.71 (1.80, 4.07) |
| Q3 vs Q1 | 5.64 (2.99, 10.64) | 5.03 (3.37, 7.52) |
| Q4 vs Q1 | 9.61 (5.11, 18.07) | 10.51 (7.00, 15.77) |
| **WWI** |  |  |
| Q2 vs Q1 | 1.84 (1.11, 3.06) | 2.11 (1.54, 2.90) |
| Q3 vs Q1 | 2.43 (1.49, 3.96) | 3.55 (2.57, 4.90) |
| Q4 vs Q1 | 3.14 (1.92, 5.14) | 5.82 (4.06, 8.34) |
| **ABSI** |  |  |
| Q2 vs Q1 | 1.04 (0.70, 1.54) | 1.29 (0.96, 1.74) |
| Q3 vs Q1 | 1.11 (0.77, 1.61) | 1.64 (1.22, 2.21) |
| Q4 vs Q1 | 1.30 (0.89, 1.91) | 1.80 (1.29, 2.49) |
| **CI** |  |  |
| Q2 vs Q1 | 2.37 (1.30, 4.30) | 2.80 (1.85, 4.23) |
| Q3 vs Q1 | 2.85 (1.61, 5.05) | 5.14 (3.42, 7.71) |
| Q4 vs Q1 | 4.31 (2.44, 7.62) | 7.62 (4.99, 11.63) |

The data is represented by OR and 95% CI.

The model adjusted for sex, race/ethnicity, level of education, smoking status, alcohol Consumption status, hypertension, and diabetes.

BRI, body roundness index; WWI, weight-adjusted waist index; ABSI, body adiposity index; CI, conicity index.

Supplementary Table 4: The association between adiposity parameters and hyperuricemia stratifed by glycemic status (with and without diabetes mellitus).

|  | Diabetes mellitus (+) | Diabetes mellitus (-) |
| --- | --- | --- |
| **BRI** |  |  |
| Q2 vs Q1 | 0.38 (0.18, 0.83) | 3.01 (2.12, 4.26) |
| Q3 vs Q1 | 0.49 (0.30, 0.82) | 4.96 (3.52, 6.99) |
| Q4 vs Q1 | * | 9.26 (6.57, 13.06) |
| **WWI** |  |  |
| Q2 vs Q1 | 2.09 (0.40, 10.87) | 2.01 (1.54, 2.62) |
| Q3 vs Q1 | 2.08 (0.43, 10.09) | 3.11 (2.38, 4.07) |
| Q4 vs Q1 | 3.59 (0.73, 17.59) | 4.34 (3.25, 5.79) |
| **ABSI** |  |  |
| Q2 vs Q1 | 0.56 (0.17, 1.86) | 1.24 (0.97, 1.58) |
| Q3 vs Q1 | 0.82 (0.27, 2.50) | 1.44 (1.13, 1.82) |
| Q4 vs Q1 | 0.95 (0.32, 2.83) | 1.56 (1.21, 2.01) |
| **CI** |  |  |
| Q2 vs Q1 | 1.48 (0.27, 8.22) | 2.76 (1.96, 3.89) |
| Q3 vs Q1 | 1.41 (0.29, 6.86) | 4.36 (3.11, 6.11) |
| Q4 vs Q1 | 2.60 (0.54, 12.55) | 6.24 (4.42, 8.82) |

The data is represented by OR and 95% CI.

The model adjusted for sex, race/ethnicity, level of education, smoking status, alcohol Consumption status, hypertension, and diabetes.

BRI, body roundness index; WWI, weight-adjusted waist index; ABSI, body adiposity index; CI, conicity index.

* The data included are too small for statistical analysis
